# Will ChatGPT’s Free Language Editing Service Level the Playing Field in Science Communication?: Insights from a Collaborative Project with Non-native English Scholars

**DOI:** 10.5334/pme.1246

**Published:** 2023-12-19

**Authors:** Lorelei Lingard, Madawa Chandritilake, Merel de Heer, Jennifer Klasen, Fury Maulina, Francisco Olmos-Vega, Christina St-Onge

**Affiliations:** 1 Centre for Education Research & Innovation, Schulich School of Medicine & Dentistry, Western University, London, Ontario, Canada; 2 Faculty of Medicine, University of Kelaniya, Sri Lanka; 3 Amsterdam UMC, Vrije Universiteit Amsterdam, Research in Medical Education, Amsterdam, Netherlands; 4 University Digestive Health Care Center, Department of Visceral Surgery, St. Clara Hospital and University Hospital Basel, Switzerland; 5 Faculty of Health, Medicine and Life Sciences, Maastricht University, Maastricht, the Netherlands; 6 Department of Public Health, Faculty of Medicine, Universitas Malikussaleh, Lhokseumawe, Aceh, Indonesia; 7 Department of Medicine and Health Profession Education Center, Universitéde Sherbrooke, Sherbrooke, Canada

## Abstract

ChatGPT has been widely heralded as a way to level the playing field in scientific communication through its free language editing service. However, such claims lack systematic evidence. A writing scholar (LL) and six non-native English scholars researching health professions education collaborated on this Writer’s Craft to fill this gap. Our overarching aim was to provide experiential evidence about ChatGPT’s performance as a language editor and writing coach. We implemented three cycles of a systematic procedure, describing how we developed our prompts, selected text for editing, incrementally prompted to refine ChatGPT’s responses, and analyzed the quality of its language edits and explanations. From this experience, we offer five insights, and we conclude that the optimism about ChatGPT’s capacity to level the playing field for non-native English writers should be tempered.

In the writer’s craft section we offer simple tips to improve your writing in one of three areas: Energy, Clarity and Persuasiveness. Each entry focuses on a key writing feature or strategy, illustrates how it commonly goes wrong, teaches the grammatical underpinnings necessary to understand it and offers suggestions to wield it effectively. We encourage readers to share comments on or suggestions for this section on Twitter, using the hashtag: #how’syourwriting?

## The Problem

Scholars writing in English as a non-native, additional language are at a profound disadvantage in the academic publishing arena. Every step of the writing process takes longer than for native English scholars, and additional steps such as translation and professional language editing may be required, adding time and resources. Such “manifold costs” perpetuate inequity in academic publishing and constrain our efforts to diversify the knowledge base [1].

Enter GenAI. Debate continues to rage about its possibilities and its problems, but a singularly positive refrain has been its potential to advance equity in science as a free language editing tool [2,3,4,5,6,7]. Large language models are being heralded as “a huge boon” (p15) [8], “tremendously helpful [for scholars] …tired of being criticized by the reviewers and editors … for not using the standard English” (p1149) [9], and even a “fire of Prometheus for non-native English-speaking researchers in academic writing” (p952) [10]. There is wide-spread optimism that they will “bridge [the] language gap, ensuring that [non-native English scholars’] invaluable research and insights are not marginalized by linguistic constraints” (p924) [11]. ChatGPT, the most popular large language model, is expected to “level the playing field, for example by removing language barriers and enabling more people to write high-quality text” (p 226) [12]. However, such claims are, to date, unsupported by systematic inquiry.

## Our Purpose

This Writer’s Craft aims to address this gap by describing a collaborative project among a writing scholar (LL) and six non-native English scholars conducting health professions education research. In this project, we developed a shared procedure for using ChatGPT as a language editor and reflecting critically on its responses. We also experimented with whether ChatGPT could be prompted to serve as a writing coach by offering precise explanations of its edits so that the writer might better apply the knowledge to future writing tasks. Our overarching aim was to provide systematic, experiential evidence about ChatGPT’s performance as a language editor and writing coach.

## Our Team

Our team includes scholars from various career stages, native language backgrounds, and writing strategies. This diversity was purposeful: we wished to explore a variety of lived experience with non-native English writing and to provide readers with a range of examples they might identify with. Our hope is that readers will see some of their own struggles and strategies in the examples we provide, thus strengthening the transferability of our insights.

MC is a professor in Sri Lanka whose native language is Sinhala. He has pursued his undergraduate and postgraduate degrees in English. He writes directly in English. He occasionally uses Grammarly for language editing assistance.

MdH is a doctoral student and medical doctor in the Netherlands whose native language is Dutch. She writes directly in English, editing her drafts with her supervisors. She sometimes uses a language editing service to help with grammar and structure. She had never used ChatGPT before.

JK is an assistant professor of surgery in Switzerland whose native language is German. She writes directly in English, editing grammar with an AI tool like Grammarly. She sometimes explains her ideas to ChatGPT and asks it to create a rough draft she can work on.

FM is a general practitioner and junior lecturer in Indonesia, and a doctoral student in the Netherlands. Her native language is Bahasa Indonesia. She writes in either Indonesian or English. She uses DeepL and Google Translate for translation, and QuillBot premium and ChatGPT3.5 for paraphrasing.

FOV is an assistant professor and anesthetist in Bogotá, Colombia. He writes either in Spanish or English, depending mostly on the nature of the project. He uses ChatGPT to generate first drafts from bullet points and then uses Grammarly to edit the results. For translations, he uses DeepL.

CStO is a professor in Canada whose native language is French. She writes directly in English, although she does some literal translations. This year she has been using writing aids such as Grammarly Pro (which rolled out Beta versions of AI to suggest changes) and ProWritingAid.

## Our Procedure

**Figure 1:**
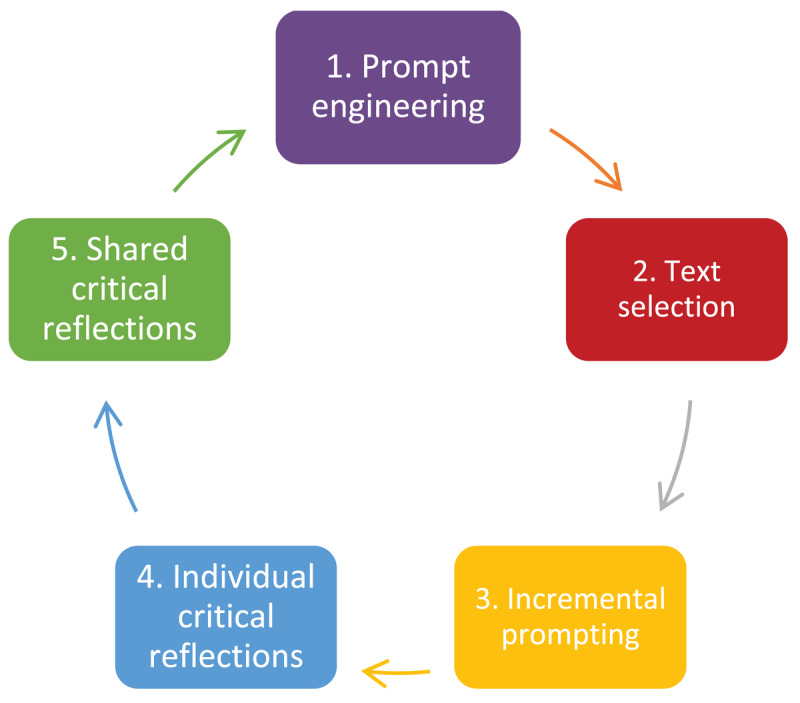
A systematic procedure for exploring ChatGPT’s free language editing capacity.

We met to discuss our aims, our experiences with ChatGPT and other AI programs, and the non-native English scholars’ current approaches to language editing their manuscripts. We chose to work with ChatGPT3.5 because it is the free version and therefore most relevant to the claims of free language editing levelling the scientific communication playing field. We shared a template for customizing instructions in our ChatGPT accounts to highlight language editing as a global purpose of our chats. Following this orienting discussion, we embarked on the following process (Figure 1).

We undertook three cycles of the process, described below, between October 11 and Nov 11, 2023.

### First cycle

**Figure 2:**
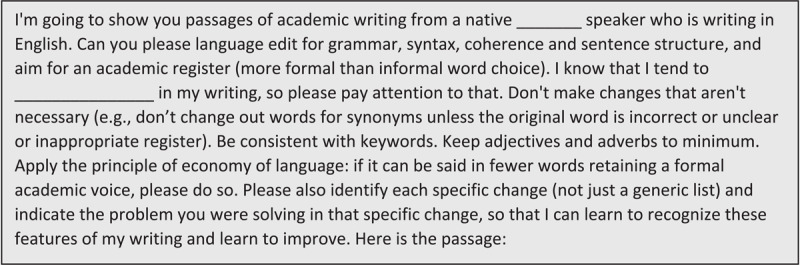
First Prompt for language editing and explanations (948 characters, ~237 tokens).

First, we discussed a draft prompt that LL had constructed using four common AI prompt components of Instructions, Context, Input Data and Output Indicator [13]. A prompt is an instruction that the user inputs into ChatGPT; optimizing the prompt quality improves the response quality [14]. In ChatGPT, the prompt-response cannot exceed 4097 tokens, or ~16,300 characters [15]. We revised the draft prompt together to enhance clarity and specificity, and to allow each author to tailor to their native language and any pre-identified writing challenges (Figure 2).

Second, we agreed that each author would select 1–2 short paragraphs of text and input that text into ChatGPT along with the revised prompt. To preserve confidentiality of unpublished data, all paragraphs were chosen from the Introduction or Discussion section of a draft paper. Third, each author engaged in incremental prompting cycles: they evaluated ChatGPT’s initial response and tinkered to improve its quality: e.g., “Please be more precise: name the change, e.g., changed the prepositional phrase. Please enumerate each change in the list; do not provide a summary note.” (FOV chatlink 1) Fourth, each non-native English author reflected individually on their chat, focusing on their sense of the quality of ChatGPT’s edits and the utility of its explanations of those edits (for an example, see supplemental Appendix A, FM reflection 1). Fifth, they shared their chat links and critical reflections with LL, who reviewed both and made her own individual, critical reflections and shared these with the group for discussion over email.

### Second cycle

**Figure 3:**
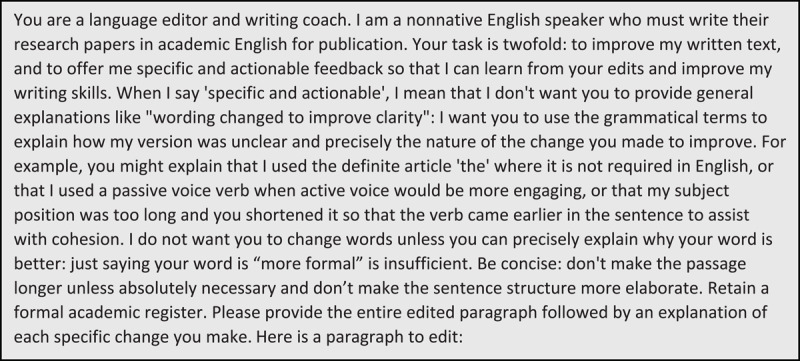
Second, revised prompt for language editing and explanations (1326 characters, ~332 tokens).

Based on our experience in the first cycle, we revised the original prompt (Figure 3) to: provide more specificity regarding the type of explanations we were seeking about the edits; offer examples; add restrictions regarding neutral changes and conciseness; and refine the parameters for its output. We also decided, if possible, to input less polished English paragraphs for editing, to see whether this affected the nature of the edits: three authors inputted less polished paragraphs in the second cycle. We then cycled individually through steps 1–4 again, and LL reviewed the chats and reflections and shared a summary for email discussion.

### Third cycle

**Figure 4:**
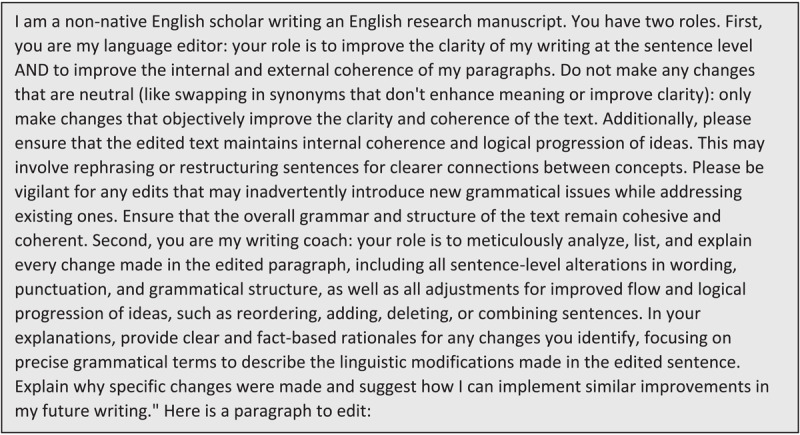
Third revised prompt for language editing and explanations (1596 characters, ~399 tokens).

Based on the results of the second cycle, LL enlisted ChatGPT’s assistance in strengthening the prompt. This involved training it on the other authors’ paragraphs from cycles 1 and 2, and incrementally asking it how to avoid errors by refining the prompt (Figure 4). Four authors used this third, revised prompt with a new paragraph.

## Our Results

Over the course of the project, the six non-native English authors put 17 paragraphs into ChatGPT for language editing, and it generated both edited versions of those paragraphs and explanations of 127 identified edits. Below we summarize our results related to using ChatGPT first as a language editor and second as a writing coach. These insights are illustrated with representative examples from our chat logs and reflections.

### ChatGPT as a language editor

ChatGPT was an enthusiastic editor, identifying between 5 and 14 edits per paragraph. Sometimes, the edited version was perceived by the author to be largely improved: one commented “In general, I am pleased with the edited version” (FM), while another reflected “I’m happy about the content, but I’m not sure exactly why I like the final output; it’s like it sounds right and nice, it makes sense, and it has a more precise and structured way of saying what I was trying to say in the first place” (FOV). Other times, authors were less positive: one reflected “To my surprise, I am disappointed in ChatGPT’s suggestions. I would only agree to 2 out of 9 changes” (JK), and another admitted to being “a bit lost”, particularly when subsequent chats generated conflicting suggestions (MdH).

As we conducted the three cycles of this project, all authors commented on the importance of looking critically at ChatGPT’s edits rather than accepting them at face value. For example, one explained that “one thing I do when I read each ChatGPT’s ‘corrected’ sentence is verify if it means what I wanted to say” (CStO). Many authors commented on the importance of retaining human judgment when using ChatGPT as an editor, but at the same time all acknowledged the difficulty of judging the quality of ChatGPT’s edits. Their reflections contained many examples of struggling to understand why ChatGPT made a particular change. Sometimes they were confused at the edits: “I think, [moreover and furthermore] have the same meaning (their role as transition words). Or are they different in their usage? I have no idea” (FM). Other times they suspected that the edits weren’t accurate: “I don’t think that’s correct in my research context. The “system” is not the same as the “framework”, in my opinion.” (FM) The frequency and quality of the edits did not substantially change in cycle two, when three authors inputted more roughly drafted paragraphs.

Part of our procedure involved LL doing an independent assessment of ChatGPT’s edits. This assessment helps to illustrate why authors might struggle to ascertain which of ChatGPT’s edits are valuable and which are not. Table 1 shows a representative original and edited paragraph, highlighted to visually reflect LL’s analysis of the edits. Four edit types are apparent: changes to verb tense or mood, changes in wording using synonyms with neutral benefit, changes to sentence construction, and improvements in phrasing for accuracy, clarity or conciseness. In LL’s assessment, the two changes in verb tense/mood have a neutral impact on the passage: the original verbs were fine. Many of the changes in wording have neutral impact as well (e.g., “considering” to “recognizing”, “regarding” to “concerning”), but many arguably weaken the passage by introducing more elaborate or assertive constructions (e.g., “growing” to “increasingly prevalent”, “worldwide” to “on a global scale”).

**Table 1:**
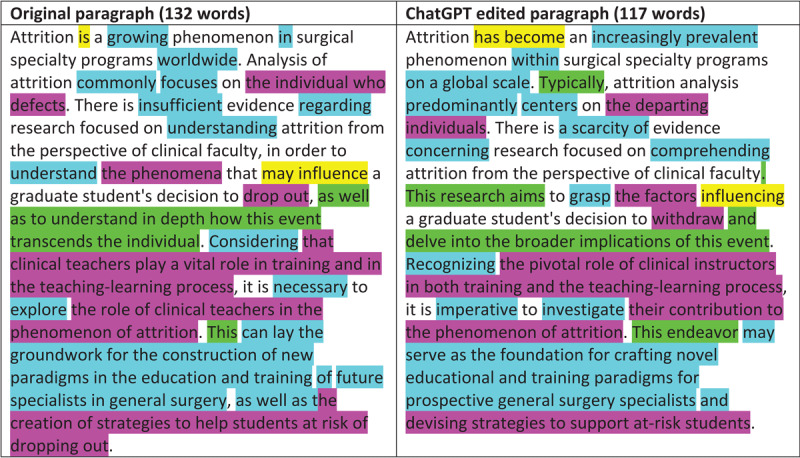
Comparison & analysis of one original and ChatGPT-edited introductory paragraph. 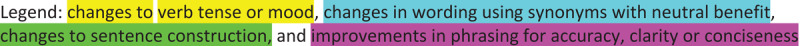

Many authors noted that ChatGPT seemed to “fancy up” their prose, but they were not always sure that this was an improvement. They also worried about retaining their own sense of voice: as one author reflected of her paragraph edits, “Regrettably” is used twice by ChatGPT, and it is not a word I would use. I would have kept “unfortunately”. Regrettably feels more familiar.” (CStO) However, even edits that were not improvements could serve as a useful signal to the writer. One author explained: “I find the ChatGPT suggestions helpful in identifying areas I’m not happy with, because that usually suggests my initial writing wasn’t sufficiently clear. … By trying to clarify, ChatGPT added words/lengthened the sentences. I would have used that as a cue to further revise, clarify, and streamline the ideas.” (CStO).

Some of ChatGPT’s edits *were* improvements. The rare instances when ChatGPT changed sentence construction, the impact was positive. In the paragraph in Table 1, for instance, one of the most impactful edits is breaking the long third sentence into two sentences. And highlighted in pink are places where, in LL’s assessment, the wording of an edit is not merely different but also better than the original. Four kinds of improvements are evident: the new text is more accurate (e.g., “the factors” instead of “the phenomenon”), more syntactically smooth (e.g., “the departing individuals” instead of “the individual who defects”), in a more appropriate register (e.g., “withdraw” instead of the more colloquial “drop out”), or more concise (e.g., “devising strategies to support at-risk students” instead of “the creation of strategies to help students at risk of dropping out”). However, comparing the blue and pink highlights in the representative paragraph in Table 1 suggests that, as often as not, ChatGPT’s edits are trading synonyms without improving the text. Our second prompt attempted, unsuccessfully, to minimize this tendency by adding the line: “I do not want you to change words unless you can precisely explain why your word is better.”

Notably, ChatGPT offered no paragraph level changes in the first cycle (such as reordering sentences or adding a topic or wrap sentence for internal coherence). This could suggest that ChatGPT interpreted the task in the prompt — ‘Can you please language edit’— as one of editing at the level of words, phrases and sentences. Our third prompt tried to address this by specifying that “your role is to improve the clarity of my writing at the sentence level AND to improve the internal and external coherence of my paragraphs”. This produced a few paragraph-level edits, but not consistently.

Finally, ChatGPT’s edits did not always target the most important issues in the original text. From LL’s perspective as a writing expert, it tended to focus on minor issues and overlook more major ones, limiting the value of its edits both for paragraph improvement and for writer development. For example, one of the more roughly drafted paragraphs we inputted in cycle two started with this long, syntactically complex sentence: “While high-quality assessment and outcomes data are key to ensuring quality feedback, training, and programs, *decisions about what to assess, how to assess, and what is high-quality data* **are fundamentally shaped by** values.” Three syntactic features impact the clarity and conciseness of this sentence: the opening subordinate clause delays the main subject; *the main subject* when it finally appears has a long, compound modifier; and **the verb** is passive. ChatGPT however, made only four minor edits to this sentence, trading “key” for “crucial”, “about” to “regarding”, “is” to “constitutes” and “shaped” to “influences”. It offered synonyms which don’t improve the sentence, while leaving the overly elaborate syntax untouched.

This tendency to ignore the most important problems in a sentence is arguably the most significant problem with ChatGPT as a language editor. In another example, 3 of the 5 sentences in the original Attrition paragraph in Table 1 used a pronoun in the main subject position (e.g., “There is…”, “This is…”, “It is…”). Because the main subject position is so important for keeping the reader on track with development of the main ideas [16], the paragraph’s coherence would be improved if those sentences were edited to put the main idea of the sentence in the subject position slot instead of a pronoun. But ChatGPT left all those constructions alone.

### ChatGPT as a writing coach

ChatGPT was highly selective in identifying the edits it made. In LL’s review of the chatlogs, she conducted an analysis of the actual edits versus the edits ChatGPT identified and found that, repeatedly, ChatGPT identified only a fraction of the edits it made. For instance, as shown in Table 2, it identified two edits to a single sentence when it had actually made ten. One of the eight it failed to identify was the most important improvement: breaking the original, compound sentence into two simple sentences.

**Table 2:** Assessment of ChatGPT’s identification and explanation of edits in a single sentence.


ORIGINAL SENTENCE	EDITED SENTENCE	CHATGPT EXPLANATION	QUALITY OF EDIT	QUALITY OF EXPLANATION

Competency-based time variable learning focuses on the transition from time-based education towards competency-based education, in which, ideally, there is time-variability for the individual to reach competencies at one’s own pace.	Competency-based time-variable learning emphasizes the shift from time-based education to competency-based education. Ideally, this approach allows for time variability, enabling individuals to attain competencies at their own pace.”	“Competency-based time variable learning” to “Competency-based time-variable learning” – Improved hyphen usage for correctness and consistency. “competencies at one’s own pace” to “competencies at their own pace” – Ensured gender-neutral language.	Variable Breaking the sentence into two is a high-quality edit. The two hyphen edits are accurate. All other edits are neutral trading of synonyms: “focuses” to “emphasizes” “transition” to “shift” “towards” to “to” “there is” to “this approach allows” “reach” to “attain” “one’s” to “their”	Incomplete: 2/10 edits are noted. Minor issues in the edit are explained, like the correction of hyphen usage, while major edits are ignored. Inaccurate: “their own” is no more gender neutral than “one’s own”


Furthermore, ChatGPT’s explanations were imprecise. It tended to offer explanations like “clarified and improved sentence structure”, leaving the author to infer the grammatical underpinnings of the change; or “provided a more formal expression”, without a convincing explanation of what is more ‘formal’ about the word it suggested. LL’s analysis could readily identify more precise explanations: e.g., ChatGPT wrote that an edit “enhances precision”, when what it had done was shift from passive to active voice. Its explanations were not only imprecise, they could also be inaccurate. For instance, not infrequently ChatGPT said an edit was “for conciseness” when the edit had more words than the original phrase. Similarly, in Table 2, the change from “one’s own” to “their own” is explained as an edit that “ensured gender-neutral language”, but the original text was already gender neutral.

Incremental prompting did not solve these problems of selective, imprecise and even inaccurate explanations of language edits. Even with incremental prompting to achieve more precise explanations, ChatGPT’s explanations remained persistently vague. Consequently, they did not offer the writer a basis for learning from this edit in order to support improvements in future piece of writing. All authors remarked on this shortcoming. One noted that “I find the feedback often too vague. In the end, I’m not sure exactly what I should change in my writing to avoid making the same mistakes again” (FOV). While another (JK), reflecting on specific ChatGPT explanations, concluded, “as a non-native with my English level, I often can’t judge the accuracy of the given description” when ChatGPT offered explanations such as that it “changed ‘despite viewing’ to ‘despite recognizing’ for more precise and accurate representation of their perception”.

Prompt engineering also did not solve these problems with ChatGPT’s explanations of its edits. Each of our prompts included stronger and more specific requests for precise grammatical explanation of every edit, with restrictions, examples and statements of role following prompt engineering best practices. Some explanations improved, but, overall, they remained selective and imprecise. Further, ChatGPT seemed to disregard aspects of the prompt even when they were explicit and specific. For instance, the first prompt included the line “I do not want you to change words unless you can precisely explain why your word is better”, but in explaining a synonym change ChatGPT admitted that its edit – “however” changed to “nevertheless” – is a neutral change: “The choice between these two words is a matter of style and preference. Both words mean the same thing.”

## Our Insights

Large language models like ChatGPT are inspiring high hopes in the research community. The results of this collaborative project suggest that this optimism needs tempering. Six scholars – with different native languages, levels of experience writing in English, and current practices for language editing their texts with and without AI – all experienced similar struggles when they tried to use ChatGPT as a language editor and a writing coach. From our experience, we offer these five insights:

**ChatGPT was not designed as a language editing tool, and it does not excel in this capacity**. It can edit the text a writer inputs, but not all of its edits are improvements. Many have a neutral impact: a trading of synonyms that doesn’t improve accuracy, clarity or conciseness. And others have a negative impact. It seems particularly prone to elaborate constructions and flowery diction and may alter the writer’s intended meaning. Most concerning, we found that ChatGPT’s edits did not prioritize the most important writing issues in a passage. If the writer already knows their writing weaknesses, they can specifically request edits of these issues and ChatGPT will comply. But if they do not have this knowledge, they cannot trust that ChatGPT has edited the main issues. Therefore, we conclude that, at this point in its development, ChatGPT3.5’s remarkable ability to *generate new text* is not matched by its ability to *edit existing text*. We recommend that non-native English writers explore other AI tools, such as Quillbot, Grammarly and ProWritingAid, that were designed for language editing. Anecdotally, our co-authors who already use these tools perceived that they often provided more useful grammar edits than ChatGPT, although these edits were also imperfect.

**Prompt engineering is important, but a simple ‘quality in, quality out’ assumption doesn’t hold**. Our efforts to increasingly refine our prompts did not consistently result in higher quality responses. ChatGPT seemed to ignore parts of our prompt, or to interpret them differently in different chats. When LL asked ChatGPT to help improve the final prompt in light of response errors, it continuously recommended refining the prompt. Eventually though, it admitted that the fault lay elsewhere: “it appears that despite the clarity of your prompt, the execution on my end was lacking. This suggests that while a well-crafted prompt is necessary for a targeted response, it is not always sufficient to guarantee that the response will meet all specified criteria due to the limitations or errors in my processing.” (See supplemental Appendix B for chatlog excerpt). The lessons we take from this are that there is no perfect, foolproof prompt, and the input/output relationship is unstable.

**Human review is essential, but non-native English writers may struggle to judge which edits are valuable**. Scholars widely agree that we must apply “skills of meticulously reviewing and adeptly editing [GenAI] outputs” [17], but what, exactly, does that mean for non-native English writers assessing ChatGPT-generated language edits? Our 5-step procedure, particularly the critical reflection steps, could assist authors in systematically approaching their language editing interactions with ChatGPT. However, our process included the reflections of a writing scholar and exchanges among our team. For writers using ChatGPT in isolation, we perceive a catch-22: the writers who would use it for language editing are the same writers who could lack the knowledge and confidence to assess its edits. All of our non-native English authors struggled with such assessments. They noted that “I become confused about what is “precise”, “concise”, “clarity”, and “clear” then? How can a non-native English writer like me define them more easily?” (FM) They reflected that “I often work with the concept of “how it sounds.” And its suggestions didn’t sound right” (JK). Similarly, authors found it challenging to know whether the edited “tone” was right: one author “realised that the tone of the language in the edited version appeared somewhat foreign to the local readership” (MC). Finally, many authors acknowledged that “a determining factor -for me- is confidence” (CStO) in assessing language edits; that confidence, however, may be lacking or undermined in ChatGPT interactions.

**ChatGPT is not an effective writing coach**. It does not identify most of its edits, and its explanations of identified edits are usually imprecise and sometimes inaccurate. It either does not have particularly robust knowledge of grammar and linguistics, perhaps reflecting limitations in its training data, or it does not access that information/vocabulary for its responses. And it is certainly not a stylish academic writer – its edits often introduce a rather stuffy, academic-speak into the text – so using it as a role model is problematic. Therefore, we conclude that ChatGPT is not a good resource for writers to learn from because its explanations are insufficient to help them recognize their recurring challenges in a passage, understand how to address them in the specific instance, and transfer this learning to future writing tasks.

**Used uncritically, ChatGPT might widen the equity gap in science communication**. ChatGPT generates responses based on predicting co-occurring words in its Western-dominated training materials [17]. As a language editor, therefore, it presents two threats: it could make texts more homogenous *and* reinforce cultural and linguistic hegemony. Writing scholars have long recognized that science writing reinforces positivist ontologies and called for writing pedagogy to include critical and hybrid genres [18]. Critical scholars have called for writing practices that dismantle conventional notions of quality in science [19].Academic literacy scholars have also warned that, in the effort to sound scholarly, writers will avoid taking risks in their writing and silence their own unique voices, such that diverse “modes of expression are revised or erased” (p1) [20]. If such erasure becomes automated in free language editing, then ChatGPT, rather than levelling the playing field in science communication, might be invisibly widening inequities. There is the possibility that unique voices in scientific writing will gain value, given that GenAI can so readily produce a homogenous default text. If this shift occurs just as non-native English writers’ voices are being homogenized by automated language editing, the leaky pipeline of publications from beyond the Global North will persist [21].

### Limitations

Our project was conducted using ChatGPT3.5 because our aim was to explore current assumptions about GenAI’s *free* language editing potential. We did not use any plug-ins, which might have altered the nature of the edits and explanations that were generated. We acknowledge that ChatGPT and other GenAI are developing quickly, and we recognize that the insights we offer here will eventually be made redundant. However, they remain critically important in the present. Many HPE scholars have not yet used GenAI technology, and, for Global South scholars in particular, we expect that their early use will involve free versions. Therefore, systematic inquiry such as our project offers is necessary to guide, and moderate, the expectations of authors, reviewers, and editors.

There are many variants and degrees of ‘non-native English drafts’, and our collaborative project cannot reflect them all. While our team includes a range of non-native English scholars in terms of career stage, native language and publishing experience, most of the paragraphs inputted were already at a reasonably good level of English in terms of grammar and syntax. While we explored whether more preliminary drafts might prompt ChatGPT to offer more meaningful edits, our limited effort in this direction was not promising.

## Conclusions

ChatGPT3.5 is not the free fire of Prometheus for non-native English scholars. As a language editor, its performance is variable and prioritizes minor writing issues. As a writing coach, its performance is poor. Our scholarly discussions should be more critically reflective of both its promise and its problems as a resource to widen global access to publishing and diversify scientific knowledge.

## Additional Files

The additional files for this article can be found as follows:

10.5334/pme.1246.s1 Supplemental Appendix A. Sample chatlog and reflection from cycle one.

10.5334/pme.1246.s2 Supplemental Appendix B. Chatlog Excerpt, Oct 28, LLingard.
