# Supplementary material for: Will ChatGPT’s Free Language Editing Service Level the Playing Field in Science Communication?: Insights from a Collaborative Project with Non-native English Scholars
Source: Perspect Med Educ. 2023 Dec 19;12(1):565–74. doi: 10.5334/pme.1246 (PMC10756157; doi:10.5334/pme.1246)
Supplement: Supplemental Appendix A. — Sample chatlog and reflection from cycle one. [file pme-12-1-1246-s1.pdf]

Supplemental Appendix A: Sample chatlog and reflection from cycle one

User

I'm going to show you passages of academic writing (from a native Indonesian speaker who is writing in English). Can you please language edit for grammar, syntax, coherence and sentence structure, and aim for an academic register (more formal than informal word choice). Don't make changes that aren't necessary (e.g., don't change out words for synonyms unless the original word is incorrect or unclear or inappropriate register). Be consistent with keywords. Keep adjectives and adverbs to a minimum. Apply the principle of economy of language: if it can be said in fewer words retaining a formal academic voice, please do so. Please also identify each specific change (not just a generic list) and indicate the problem you were solving in that specific change so that I can learn to recognize these features of my writing and learn to improve. Here is the passage:

Primary care, as mentioned by the World Health Organization, involves essential care for the communities and is the first element of a continuing healthcare process. It is also the backbone of a rational health services system. More critically, primary care is the leader in providing health care to underserved areas. As a result, primary care requires a strong physician leader. Additionally, according to the literature, a qualified physician leader plays a key role in the success and improvement of primary care, either in formal or informal roles in their healthcare settings. In fact, physicians, although expected to be leaders, rarely receive training on how to be effective leaders on a broader organizational or systems level, as a result, rural municipalities frequently have no other choice except to employ physicians who lack such qualifications. In South Africa, for instance, using the Delphi technique, they discovered that lack of necessary training and poor leadership skills were key drivers of problems in a broader rural health system, such as uninformed decision-making, a lack of urgency in handling crises, a poor work ethic, poor work relations, and low staff morale. Also, from an education perspective, many medical schools lack formal leadership curricula. This may reflect time constraints in the existing curriculum, limited resources, beliefs that leadership cannot be taught, and a lack of consensus about the content of leadership courses.

Edited Paragraph by ChatGPT

Primary care, as highlighted by the World Health Organization, encompasses essential community care and serves as the foundational component of an ongoing healthcare process. It forms the cornerstone of a well-structured healthcare system. Moreover, primary care plays a pivotal role in delivering healthcare to underserved regions. Consequently, primary care necessitates a proficient physician leader. Furthermore, in accordance with the literature, a competent physician leader plays a pivotal role in enhancing the success of primary care, whether in formal or informal capacities within healthcare settings. In reality, while physicians are expected to assume leadership roles, they seldom receive training in effectively leading at a broader organizational or systemic level. Consequently, rural municipalities often have no alternative but to hire physicians lacking such qualifications. For instance, in South Africa, through the application of the Delphi technique, it was found that the absence of essential training and deficient leadership skills were prominent factors contributing to challenges in the broader rural healthcare system. These issues encompassed uninformed decision-making, a dearth of urgency in crisis management, subpar work ethics, compromised work relationships, and diminished staff morale. Furthermore, from an educational standpoint, numerous medical schools lack structured leadership curricula. This deficiency may be attributed to time constraints in the current curriculum, resource limitations, the belief that leadership is unteachable, and a lack of consensus regarding the content of leadership courses.

Here are the edits and explanations for each change made in the passage:

1. Original: Primary care, as mentioned by the World Health Organization, involves essential care for the communities and is the first element of a continuing healthcare process.
   Edited: Primary care, as highlighted by the World Health Organization, encompasses essential community care and serves as the foundational component of an ongoing healthcare process. Explanation: Replaced "involves" with "encompasses" for precision and replaced "and is" with "and serves as" for conciseness.
2. Original: It is also the backbone of a rational health services system.
   Edited: It forms the cornerstone of a well-structured healthcare system.
   Explanation: Replaced "backbone" with "cornerstone" for a more formal and concise expression.
3. Original: More critically, primary care is the leader in providing health care to underserved areas. Edited: Moreover, primary care plays a pivotal role in delivering healthcare to underserved regions. Explanation: Enhanced formality by replacing "More critically" with "Moreover" and improved sentence structure for clarity.
4. Original: As a result, primary care requires a strong physician leader.
   Edited: Consequently, primary care necessitates a proficient physician leader.
   Explanation: Substituted "requires" with "necessitates" for a more formal tone and clarity.
5. Original: Additionally, according to the literature, a qualified physician leader plays a key role in the success and improvement of primary care, either in formal or informal roles in their healthcare settings. Edited: Furthermore, in accordance with the literature, a competent physician leader plays a pivotal role in enhancing the success of primary care, whether in formal or informal capacities within healthcare settings.
   Explanation: Improved clarity and formality by restructuring the sentence.
6. Original: In fact, physicians, although expected to be leaders, rarely receive training on how to be effective leaders on a broader organizational or systems level, as a result, rural municipalities frequently have no other choice except to employ physicians who lack such qualifications.
   Edited: In reality, while physicians are expected to assume leadership roles, they seldom receive training in effectively leading at a broader organizational or systemic level. Consequently, rural municipalities often have no alternative but to hire physicians lacking such qualifications.
   Explanation: Enhanced clarity and formality. Restructured the sentence for better flow.
7. Original: In South Africa, for instance, using the Delphi technique, they discovered that lack of necessary training and poor leadership skills were key drivers of problems in a broader rural health system, such as uninformed decision-making, a lack of urgency in handling crises, a poor work ethic, poor work relations, and low staff morale.
   Edited: For instance, in South Africa, through the application of the Delphi technique, it was found that the absence of essential training and deficient leadership skills were prominent factors contributing to challenges in the broader rural healthcare system. These issues encompassed uninformed decision-making, a dearth of urgency in crisis management, subpar work ethics, compromised work relationships, and diminished staff morale.
   Explanation: Clarified the subject and object of the sentence and improved the structure for academic formality.
8. Original: Also, from an education perspective, many medical schools lack formal leadership curricula. This may reflect time constraints in the existing curriculum, limited resources, beliefs that leadership cannot be taught, and a lack of consensus about the content of leadership courses.
   Edited: Furthermore, from an educational standpoint, numerous medical schools lack structured leadership curricula. This deficiency may be attributed to time constraints in the current curriculum, resource limitations, the belief that leadership is unteachable, and a lack of consensus regarding the content of leadership courses.

Explanation: Improved formality and made the sentence more concise and clear.

Co-author reflections on the language edited by ChatGPT

1. In several points i.e. points 1, 5, 6, 7, and 8, I did not see anything that differed from my sentences. However, at first glance, they seem sophisticated, academic, and formal.
2. Point 2, I prefer to use the term 'the backbone' instead of 'the cornerstone' since, as far as I know, the backbone refers to physical features, but I am not sure what 'the cornerstone' refers to. So, I think, 'the backbone' is a 'stronger' term than 'the cornerstone' in the context of my research.
3. Point 3, I have even seen an article where 'more critically' is placed at the beginning of the sentence. I did not consider them as 'transition' but rather emphasized that primary care is important in providing health services for underserved areas. Nonetheless, I agreed with "…plays a pivotal role...".
4. In accordance with point 1 (replaced "involves" with "encompasses"), point 4 (substituted "requires" with "necessitates"), and point 5 (replaced "roles" with "capacities"), I did not find them to be too different. However, they may differ in terms of the level of necessity (I guess). I didn't fully understand how and in what context to use them. Personally, I, however, believe that the readers comprehend the meaning of these sentences.
5. Point 4, in rural and remote contexts, we need a 'strong physician leader' rather than a 'proficient physician leader', because in these settings we require a physician leader with qualities beyond a set of clinical skills. They must be a physician who has personal qualities such as determination, resilience, and motivation.

In general, I am pleased with the edited version because it also encouraged me to learn more about how to write scientific writing to become precise, concise, formal, academic, more clarity, etc. However, at the same time, I also become confused about what is precise, concise, clarity, and clear then? How can a non-native English writer like me define them more easily?

It is more important, in my opinion, to provide writing that is reader-friendly. This fits also my habits and writing style i.e. concise and tend to write simple sentences. Character and culture also influence the way I write my ideas. However, I eventually found that, as a qualitative researcher, I must learn how to write with 'flow' in mind and deliver it with 'appropriate polish'. I realized that, in the qualitative study, even simple things if written down in depth and detail will be something valuable.

Like a pilot, 'flight hours' for writers are also important. Writing English academic articles is a 'new' experience for me (because I previously published academic articles in Indonesian). I started ±3.5 years ago (during my PhD journey). So, I would say: "I am struggling in English writing". Personally, writing in English is a long process and time-consuming for me therefore, I must be patient with the process.
